# Pros and cons of navigated versus conventional total knee arthroplasty—a retrospective analysis of over 2400 patients

**DOI:** 10.1007/s00402-021-03834-y

**Published:** 2021-02-23

**Authors:** Matthias Meyer, Tobias Renkawitz, Florian Völlner, Achim Benditz, Joachim Grifka, Markus Weber

**Affiliations:** Department of Orthopaedic Surgery, Regensburg University Medical Center, Asklepios Klinikum Bad Abbach, Kaiser-Karl V.-Allee 3, 93077 Bad Abbach, Germany

**Keywords:** Total knee arthroplasty, Navigation, Alignment, Outcome, Complications, Responder

## Abstract

**Introduction:**

Because of the ongoing discussion of imageless navigation in total knee arthroplasty (TKA), its advantages and disadvantages were evaluated in a large patient cohort.

**Methods:**

This retrospective analysis included 2464 patients who had undergone TKA at a high-volume university arthroplasty center between 2012 and 2017. Navigated and conventional TKA were compared regarding postoperative mechanical axis, surgery duration, complication rates, one-year postoperative patient-reported outcome measures (PROMs) (WOMAC and EQ-5D indices), and responder rates as defined by the criteria of the Outcome Measures in Rheumatology and Osteoarthritis Research Society International consensus (OMERACT-OARSI).

**Results:**

Both navigated (1.8 ± 1.6°) and conventional TKA (2.1 ± 1.6°, *p* = 0.002) enabled the exact reconstruction of mechanical axis. Surgery duration was six minutes longer for navigated TKA than for conventional TKA (*p* < 0.001). Complication rates were low in both groups with comparable frequencies: neurological deficits (*p* = 0.39), joint infection (*p* = 0.42 and thromboembolic events (*p* = 0.03). Periprosthetic fractures occurred more frequently during conventional TKA (*p* = 0.001). One-year PROMs showed excellent improvement in both groups. The WOMAC index was statistically higher for navigated TKA than for conventional TKA (74.7 ± 19.0 vs. 71.7 ± 20.7, *p* = 0.014), but the increase was not clinically relevant. Both groups had a similarly high EQ-5D index (0.23 ± 0.24 vs. 0.26 ± 0.25, *p* = 0.11) and responder rate (86.5% [256/296] vs. 85.9% [981/1142], *p* = 0.92).

**Conclusion:**

Both methods enable accurate postoperative leg alignment with low complication rates and equally successful PROMs and responder rates one year postoperatively.

**Level of evidence:**

III. Retrospective cohort study.

## Introduction

Total knee arthroplasty (TKA) is a frequently conducted and effective surgical method for relieving pain and restoring function in patients with severe osteoarthritis [1, 2]. The frequency of knee replacement surgery increased on average by 40% in countries belonging to the Organization for Economic Co-operation and Development (OECD) between 2007 and 2017 [2]. A further increase can be expected in the course of demographic change [3].

Accurate restoration of mechanical axis is considered an objective quality feature in TKA because it improves implant survivorship [4, 5] and correlates with clinical outcome [6–8]. Imageless navigation systems for computer-assisted TKA were developed in the 1990s to facilitate proper leg alignment and implant positioning [9]. Since then, the use of imageless navigation systems has been discussed among experts [10–16]. Although some authors did not find any differences in postoperative radiological and clinical parameters between navigated and conventional TKA [10–12], other authors found improvements in leg alignment and even in functional results after the use of navigation systems [13–15]. Imageless navigation requires longer surgery durations [14, 17, 18], partly because of the necessary insertion of Schanz screws to fix the reference arrays for optical navigation. Some authors have described an increased risk of periprosthetic fractures and nerve injuries in connection with the insertion of Schanz screws [19, 20], whereas others have reported a reduced complication rate for navigated TKA [21]. However, the comparability of these studies is restricted due to the application of different postoperative treatment protocols [13, 15, 17] or a limited number of patients [12, 14, 18].

Therefore, navigated and conventional TKA were analyzed with regard to the accuracy of mechanical axis restoration, surgery duration, complication rates, and patient-oriented outcome measures (PROMs) one year postoperatively in a single center study of 2543 TKAs conducted at a high-volume arthroplasty department. It was hypothesized that navigated and conventional TKA would differ with regard to postoperative mechanical leg alignment, surgery duration, complications, and patient-reported outcome measures one year postoperatively.

## Methods

This retrospective analysis was based on the joint registry established at our department. The study was approved by the local Ethics Commission (18-927-104).

**Fig. 1:**
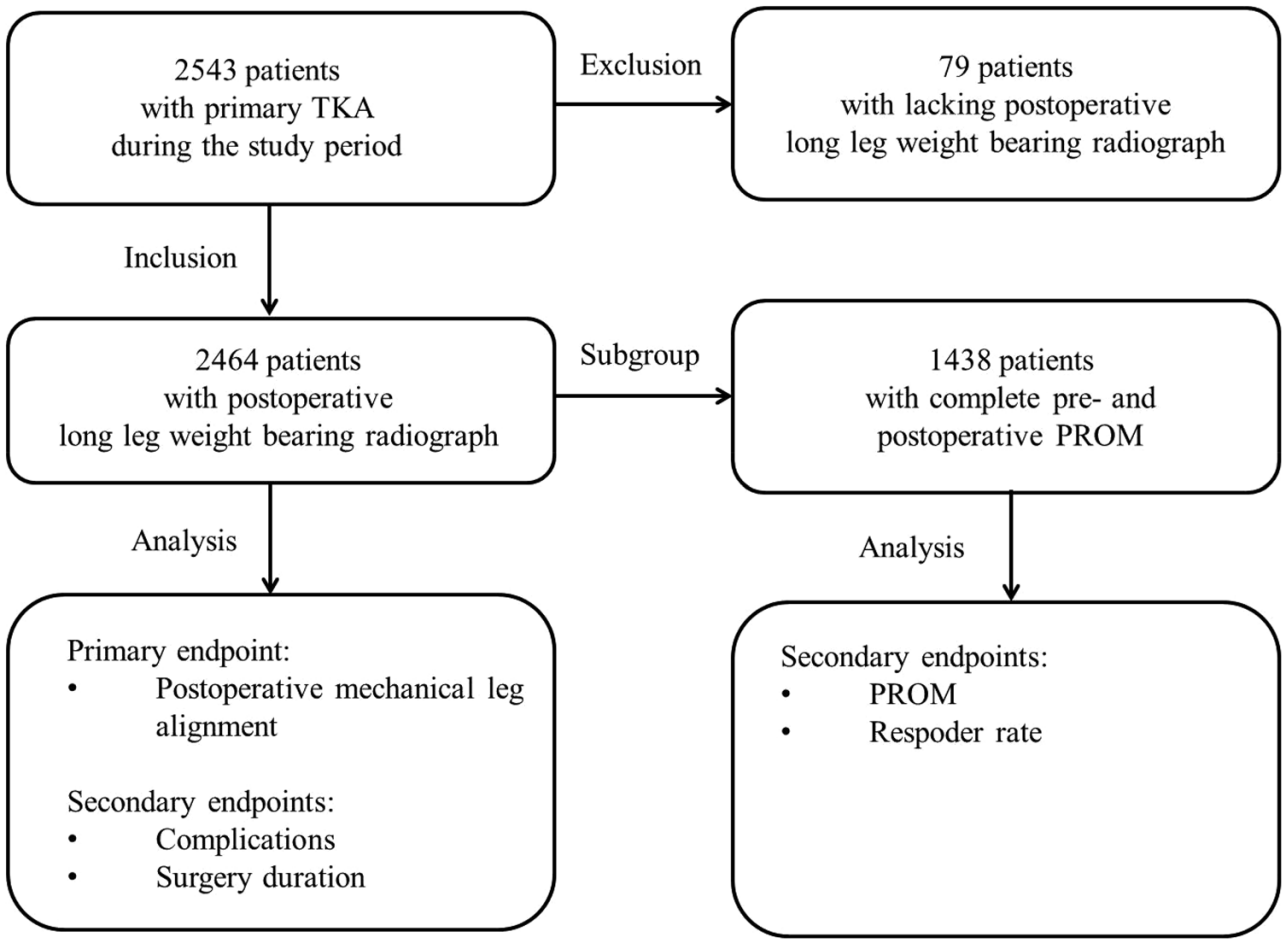
Study flow diagram

From the database, all patients who had undergone TKA because of primary or secondary osteoarthritis of the knee and who had received a postoperative long leg radiograph were included. Patients undergoing revision surgery and patients with lacking postoperative long leg radiograph were excluded. A total of 2464 patients met the inclusion criteria. Pre-operative and postoperative patient-reported outcome data were only available for a subgroup of 1438 patients (Fig. 1). The characteristics of the study group are shown in Table 1.

**Table 1:** Anthropometric characteristics of the study group

*N* = 2464	Conventional TKA	Navigated TKA	*p* value
Number of patients	*N* = 555	*N* = 1909	
Age (years)	68.1 ± 9.5	68.2 ± 9.4	0.82
Sex (women)	65.0% (361)	60.9% (1163)	0.09
ASA-Class 1	6.5% (36)	8.9% (170)	0.07
ASA-Class 2	48.6% (270)	51.9% (991)	0.19
ASA-Class 3	44.7% (248)	38.7% (739)	0.01
ASA-Class 4	0.2% (1)	0.5% (9)	0.29
Length of hospital stay (d)	9.7 ± 2.1	9.6 ± 2.9	0.37

Values of categorical data are given as relative and absolute frequencies, values of quantitative data are given as mean (standard deviation). *TKA* total knee arthroplasty, *ASA* American Society of Anesthesiologists

**Fig. 2:**
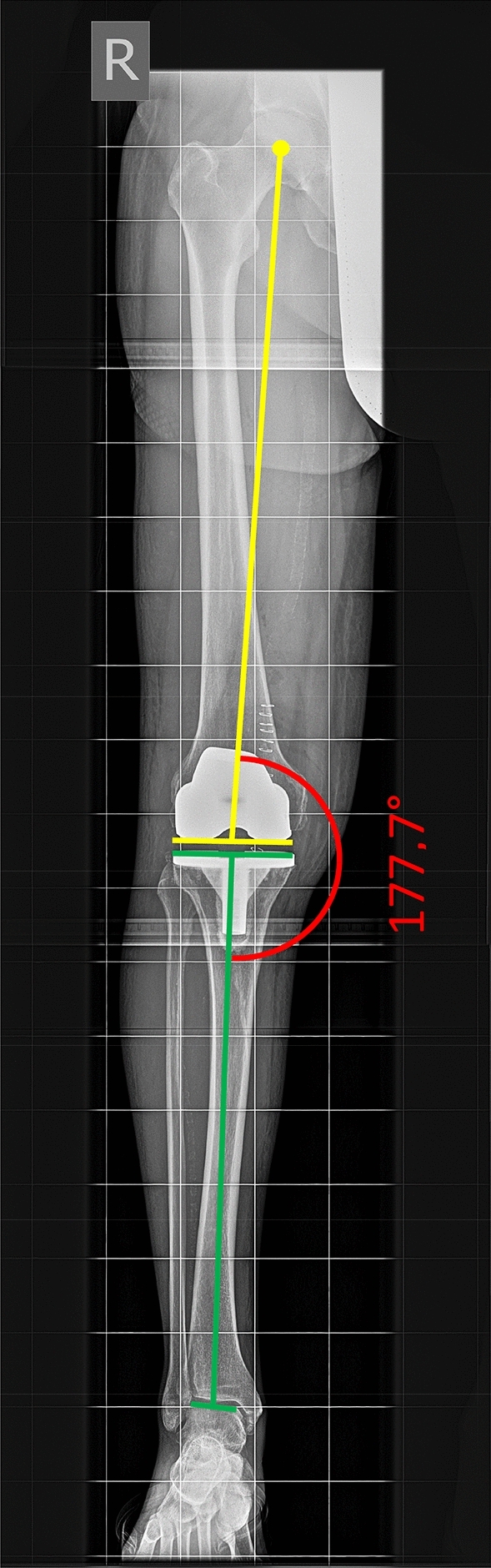
Postoperative measurement of mechanical axis on a long-leg weight-bearing radiograph

All operations took place at a single Department of Orthopedic Surgery of a University Medical Center, between 2012 and December 2017. Conventional TKA was compared to navigated TKA. Each surgical intervention involved the application of a tourniquet and the same implant system inserted by means of a medial parapatellar approach (PFC Sigma®, fixed bearing, cemented, DePuy, Warsaw, IN, USA).. All patients received patellar resection arthroplasty. Patellar resurfacing was not performed. The choice of surgical method, i.e. conventional or navigated TKA, depended on availability of a navigation system. The use of navigation was limited as only three of four operation theatres were equipped with a navigation system and due to limited availability of navigation instruments. If a navigation system was not available, conventional TKA was performed. Conventional TKA consisted of a ‘measured resection’ workflow with extramedullary alignment of tibial resection and intramedullary alignment of femoral resection. The angle between the anatomical and mechanical femur axis (AMA) was determined by means of a pre-operative long-leg weight-bearing radiograph. Navigated TKA was conducted with an imageless navigation system (BrainLAB, Munich, Germany). After the registration of the bony landmarks and the mechanical axis, navigation-guided tibial resection was conducted perpendicular to the mechanical axis of the tibia. Similarly, distal femoral resection was carried out with the assistance of the navigation system perpendicular to the femoral mechanical axis. Correct restoration of mechanical axis was controlled with trial components and recorded by the navigation system (Fig. 2). After implantation of the permanent components, mediolateral and sagittal stability of the prosthesis was assessed clinically. All surgeons used both the techniques and were equally trained and skilled in navigation and conventional implantation. All operations were performed or supervised by a senior arthroplasty surgeon. According to our institutional standard protocol, all patients were allowed immediate full weight bearing and free range of motion, depending on pain and swelling.

**Fig. 3:**
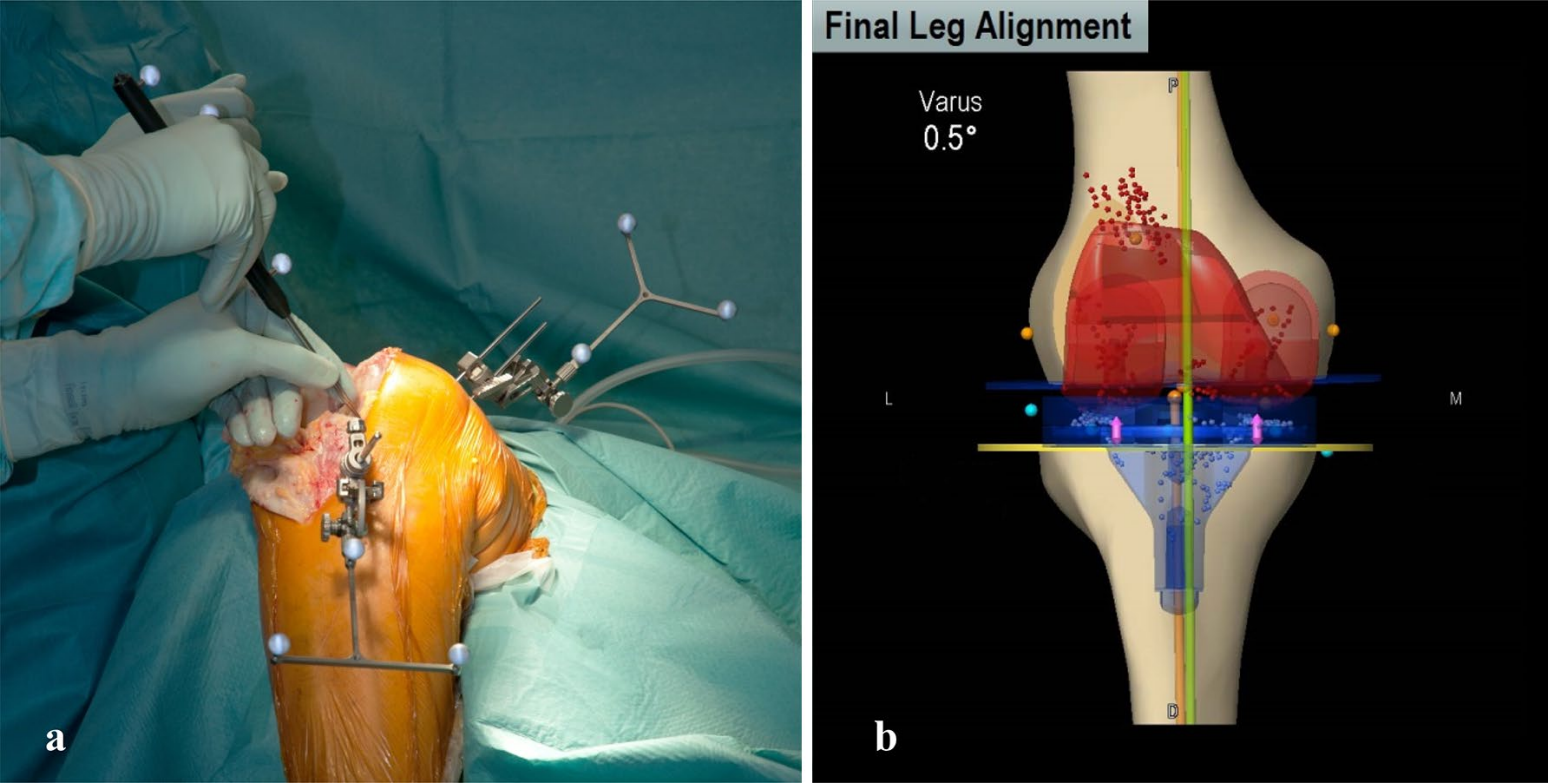
Reference arrays (**a**) and final leg alignment (**b**) in navigated TKA

Radiographic images were evaluated according to the method developed by Moreland et al. [22] using postoperative long-leg weight-bearing radiographs. To ensure correct measurement, proper limb rotation was verified by fibular overlap [23]. Femoral mechanical axis was determined by drawing a line from the center of the femoral head to the center of the femoral component. For determination of tibial mechanical axis, a line was drawn from the center of the tibial component to the center of the ankle. The angle between femoral and tibial mechanical axis was measured (Fig. 3). All measurements were made by independent observers using the same medical planning software (MediCAD®, Hectec, Landshut, Germany).

Data from the institutional joint registry, included patient age, sex, the American Society of Anesthesiologists (ASA) score, use of a navigation system, surgery duration, and complication rates. Complications included intraoperative fractures, deep joint infections, thromboembolic events and neurological deficits. Fractures were verified with postoperative X-ray. Thromboembolic events were diagnosed by color-coded duplex sonography or thoracic computed tomography. Postoperative neurological deficits were confirmed by consultation with a neurologist. Deep joint infections were defined by the German national nosocomial infections surveillance system [24].

The Western Ontario and McMaster Universities Arthritis index (WOMAC) [25] and the Euro-Qol 5D-5L index (EQ-5D) [26] were assessed preoperatively and one year postoperatively. Outcome data beyond one year postoperatively were not available in our joint registry. The WOMAC index is an internationally widely used tool for an outcome evaluation after total joint replacement [27]. In addition to the WOMAC index, patients were asked about the intake of analgesics for pain relief in the affected joint. Respondents were asked to classify the current use of analgesics according to frequency into five categories (‘always’, ‘often’, ‘sometimes’, ‘occasionally’, and ‘never’). For statistical analysis, categories were converted into scores between 4 (corresponding to ‘never’) and 0 (corresponding to ‘always’). Finally, raw scores were normalized to a scale between 0 (‘worst’) and 100 (‘best’). The EQ-5D index is a widely applied and validated tool for health assessment for evaluating health according to the dimensions of mobility, independence, care, ordinary activities, pain, discomfort, anxiety, and depression [26, 27]. Responders were differentiated from non-responders by means of the criteria of the Outcome Measures in Rheumatology and Osteoarthritis Research Society International consensus (OMERACT-OARSI) [28]. The OMERACT-OARSI criteria identify patients as responders after TKA if the WOMAC index shows an improvement in pain or function, either relatively by at least 50% or absolutely by at least 20 points. Alternatively, patients are defined as responders if two of the following criteria are met: improvement of the pain subscore by at least 20% and at least 10 points, improvement of the function subscore by at least 20% and at least 10 points, or improvement in the global index by at least 20% and at least 10 points [27].

### Statistics

The primary endpoint postoperative mechanical leg alignment was investigated by means of a power calculation. Based on the results of a previous study [29], the expected effect size of mechanical axis deviation was conservatively set to 0.25. A sample size of 275 patients in each group achieved a power of 90% using two-sample t test (nQuery Advisor 7.0, Statistical Solutions Ltd, Cork, Ireland). Secondary endpoints were surgery duration, complications, and PROMs one year postoperatively (WOMAC and EQ-5D indices).

For statistical analysis, continuous data are presented as mean ± standard deviation. Group comparisons were conducted with a two-sided t test. Categorical values are given as counts and percentages and were compared between groups with the Fisher exact test. The primary endpoint was tested on a significance level of 5%. For all secondary endpoints, the significance levels were adjusted according to Bonferroni [30]. IBM SPSS Statistics 22 (SPSS Inc., Chicago, IL, USA) was used for analysis.

## Results

**Fig. 4:**
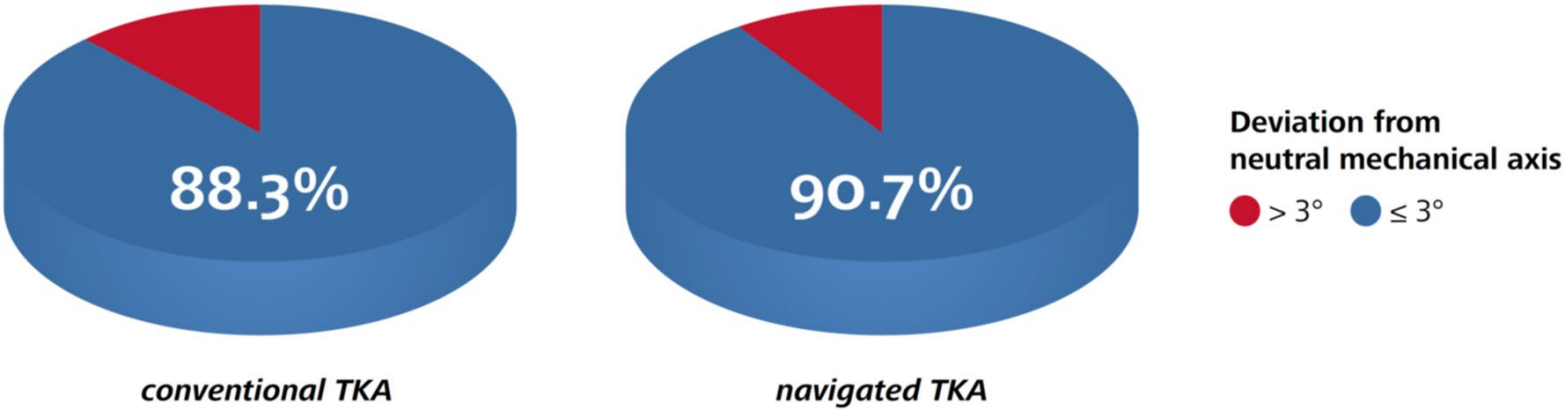
Proportions of patients with postoperative mechanical axis within and outside ± 3° deviation from neutral leg alignment after conventional and navigated TKA

Mean deviation from neutral mechanical axis after conventional TKA was 2.1 ± 1.6° versus 1.8 ± 1.6° after navigated TKA (95% CI of the difference 0.1° to 0.4°, *p* = 0.002). The proportion of patients with postoperative mechanical axis within ± 3° deviation from neutral leg alignment was 88.3% (490/555) in the conventional group compared to 90.7% (1731/1909) in the navigated group (*p* = 0.86, Fig. 4).

Mean surgery duration of 75.6 ± 23.5 min in the conventional group was six minutes shorter than in the navigated group with 81.7 ± 23.2 min (95% CI of the difference − 8.3 to 4.0 min, *p* < 0.001).

Complications were rare in both groups (Table 2). The frequency of complications was comparable between the two groups: neurological deficits (0.5% [3/555] vs. 0.3% [5/1909], *p* = 0.39), joint infection (1.1% [6/555] vs. 0.7% [14/1909], *p* = 0.42), and thromboembolic events (0.7% [5/555] vs. 0.1% [2/1909], *p* = 0.03). Periprosthetic fractures occurred more frequently during conventional TKA than during navigated TKA (1.1% [6/555] vs. 0.1% [1/1909], *p* = 0.001).

**Table 2:** Complication rates for conventional and navigated TKA

TKA	Conventional TKA	Navigated TKA	*p* -value
Intraoperative fractures	1.1% (6/555)	0.1% (1/1909)	0.001
Thrombosis	0.7% (4/555)	0.1% (2/1909)	0.03
Neurological deficits	0.5% (3/555)	0.3% (5/1909)	0.39
Joint infection	1.1% (6/555)	0.7% (14/1909)	0.42

Values of categorical data are given as relative and absolute frequencies. *TKA* total knee arthroplasty

**Fig. 5:**
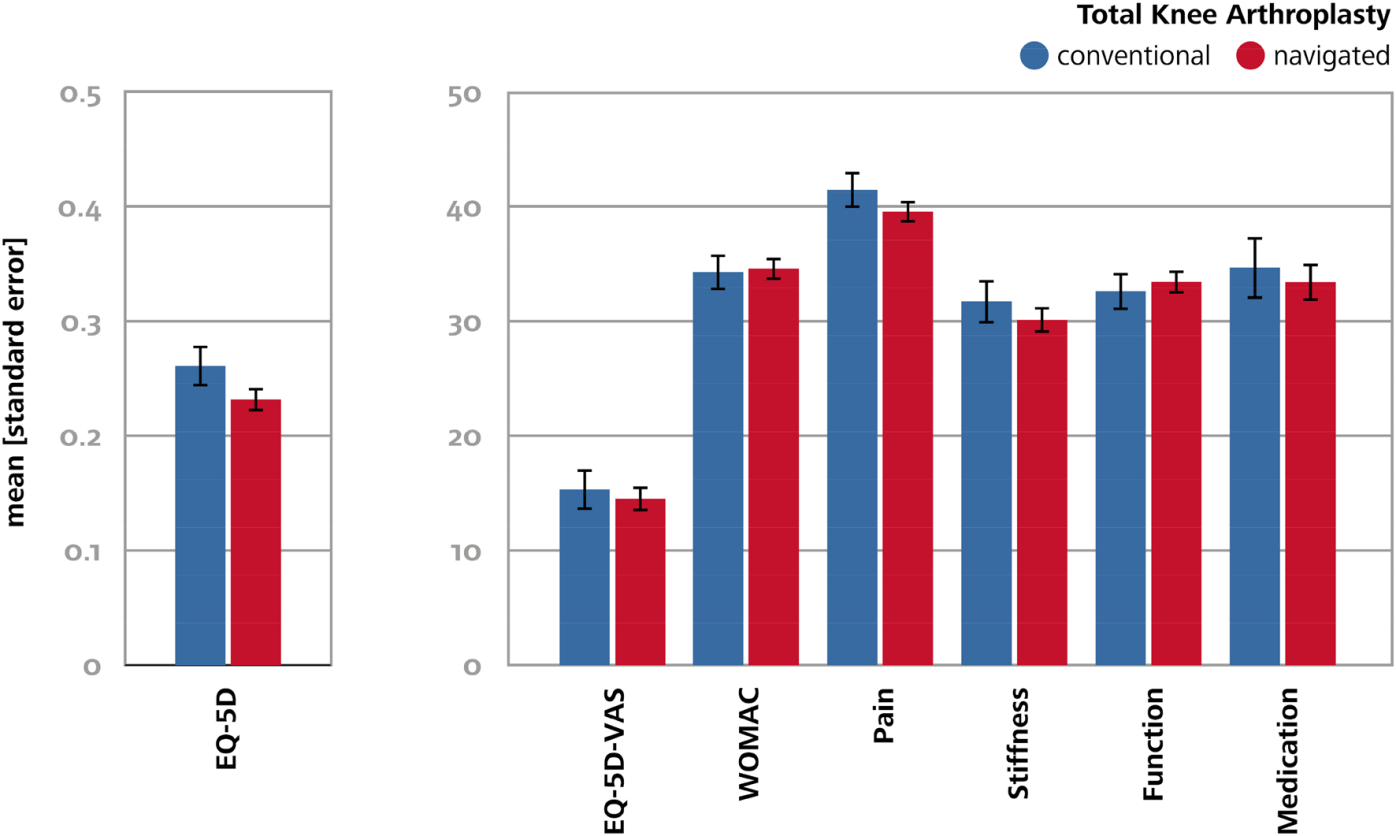
Extent of improvement with regard to Euro-Qol 5D-5L (EQ-5D), Euro-Qol 5D-5L VAS (EQ-5D-VAS) Western Ontario, and the McMaster Universities Arthritis Index (WOMAC), and use of analgesic medication one year after conventional and navigated TKA compared to pre-operative baseline values

With regard to PROMs, both groups had comparable preoperative baseline values and showed high improvement in the WOMAC index as well as in the EQ-5D index and in EQ-5D-VAS one year postoperatively (Fig. 5). Mean WOMAC index one year postoperatively was higher in the navigated group than in the conventional group (74.7 ± 19.0 vs. 71.7 ± 20.7, *p* = 0.01), but the difference was comparable in relation to pre-operative values (34.5 ± 20.2 vs. 34.2 ± 22.1, *p* = 0.85). Improvement in the EQ-5D index in relation to preoperative values was similar in both groups (0.23 ± 0.24 vs. 0.26 ± 0.25, *p* = 0.11).

Mean EQ-5D-VAS one year postoperatively was statistically higher in the navigated group than in the conventional group (69.4 ± 19.9 vs. 66.5 ± 22.1, *p* = 0.03). Evaluation of subscores showed better postoperative function as the reason for the higher postoperative WOMAC index in the navigated group (*p* = 0.01). The two groups did not show any significant differences in stiffness and pain (Table 3) and had a comparably low rate of analgesic medication one year postoperatively (75.9 ± 32.3 vs. 74.5 ± 32.4, *p* = 0.47).

**Table 3:** Western Ontario and McMaster Universities Arthritis Index (WOMAC), Euro-Qol 5D-5L (EQ-5D), Euro-Qol 5D-5L VAS (EQ-5D-VAS) and use of analgesic medication before and one year after conventional and navigated TKA

TKA		EQ-5D Preop	EQ-5D Postop	EQ-5D-VAS Preop	EQ-5D-VAS Postop	WOMAC PREOP	WOMAC Postop	Pain Preop	Pain Postop	Stiffness Preop	Stiffness Postop	Function Preop	Function Postop	Medication Preop	Medication Postop
Conv	Mean	0.54	0.80	51.9	66.5	39.3	71.7	35.7	75.8	38.7	69.3	40.1	70.7	39.5	74.5
	SD	0.22	0.21	19.3	22.1	16.2	20.7	17.6	21.4	23.1	23.1	17.3	21.3	37.4	32.4
Nav	Mean	0.56	0.81	52.8	69.4	39.4	74.7	36.5	77.7	39.1	70.4	40.2	74.2	39.8	75.9
	SD	0.21	0.20	20.3	19.9	16.2	19.0	17.6	19.5	22.3	21.5	17.4	19.8	37.2	32.3
*p* value		0.22	0.38	0.48	0.03	0.92	0.01	0.46	0.14	0.80	0.41	0.92	0.01	0.90	0.47

Values of quantitative data are given as mean (*SD* standard deviation). *TKA* total knee arthroplasty, *Conv* conventional, *Nav* navigated, *preop* preoperative, *postop* postoperative

The responder rate as defined by the OMERACT-OARSI criteria one year postoperatively was comparably high in both groups with 86.5% (256/296) in the conventional group and 85.9% (981/1142) in the navigated group (*p* = 0.92).

## Discussion

In the course of demographic change, the prevalence of degenerative joint diseases can be expected to increase in the near future [3]. At the same time, older people tend to be more active and mobile nowadays than previous generations [31]. In this context, total knee arthroplasty is a proven effective curative treatment for severe osteoarthritis of the knee in terms of pain relief, functional improvement, and enhancement of quality of life [1]. Accurate restoration of mechanical axis is considered to be one of the key factors for successful TKA because it affects implant survival [4, 5] and correlates with clinical outcome [6–8]. The current study showed that mechanical axis can be successfully restored by means of both navigated and conventional TKA. Both techniques appear to be safe treatment options with low complication rates and—according to PROMs—excellent clinical outcome one year postoperatively. Mechanical axis was exactly restored in both groups with a mean deviation from neutral alignment (straight leg) of 2°. The restoration of mechanical axis was on average 0.3° more accurate in the navigated group than in the conventional group (*p* = 0.002). Although statistically significant, the mean difference of 0.3° between navigated and conventional TKA is considered clinically irrelevant. Accuracy was comparable in both groups with a standard deviation of 1.6°. In the literature, a majority of studies state that mechanical leg axis can be more accurately restored by the intra-operative use of a navigation system [13, 14]. Regardless of absolute values, the reconstruction of mechanical axis within a range of ± 3° is considered an objective quality feature because biomechanical investigations found positive correlations between accurate mechanical alignment and implant survival [4, 5, 32, 33]. Meta-analyses found malalignment > 3° in 9.0–12.1% after navigated TKA versus 28.7–31.8% after conventional TKA [13, 34]. In our study, alignment could be restored within ± 3° deviation from neutral mechanical axis in 88.3% of patients in the conventional group compared to 90.7% in the navigation group. Therefore, conventional TKA performed nearly equally well as navigated TKA in terms of alignment reconstruction. This fact may be due to the high-volume setting in our clinic as well as a possible selection bias. However, there is an increasing evidence that small deviations from neutral mechanical alignment do not appear to impact implant survival, postoperative complication rates or PROMs after TKA at short-term follow-up, especially if alternative alignment strategies are applied [35].

In our study, mean surgery duration was six minutes longer for navigated TKA than for conventional TKA. Although this longer surgery duration for navigated TKA is consistent with literature reports, most publications describe a higher increase in surgery duration of 12–15 min on average [17, 18, 36]. We assume that the minor extension of surgery duration in our study was due to our standardized navigation workflow in a high-volume setting. The type of navigation system may also have an influence on surgery duration. Under these conditions, navigated TKA may be carried out with a minimal increase in surgery duration. In terms of complications, both navigated and conventional TKA proved to be safe. Complications were generally rare in both groups. Navigated TKA requires femoral and tibial insertion of Schanz screws to fix the reference arrays for optical navigation (Fig. 3). Periprosthetic fractures, nerve injuries, and infections caused by these screws had been described in the literature [19, 20], but could not be confirmed by our investigations. Surprisingly, more fractures occurred in the conventional group than in the navigation group. The frequency of nerve injuries and infections was equally low in both the groups. Thromboembolic events occurred more frequently in the conventional group, but accumulation was statistically not significant after Bonferroni adjustment. Although thromboembolic events due to the intramedullary alignment of femoral resection have been described, no clear evidence of a correlation between navigation and less thromboembolic events has been reported in the literature so far [37, 38].

Regarding PROMs, no relevant difference between the conventional and the navigated group could be observed one year postoperatively. Both groups showed excellent improvement in the WOMAC and EQ-5D indices. Although the global mean value of the WOMAC index one year postoperatively was higher in the navigated group than in the conventional group, the extent of improvement was not clinically relevant. These findings are consistent with the existing literature as the majority of published studies and meta-analyses of PROMs after TKA also did not show any relevant differences between navigated and conventional implantation [36, 39]. Accordingly, the proportion of responders in our study defined by the OMERACT-OARSI criteria was comparable with a responder rate of approximately 86% in both groups. This percentage corresponds with results of other authors who described 8–25% of dissatisfied patients after TKA [1, 40]. In a recently published meta-analysis comparing kinematic and mechanical alignment in TKA, PROMs even favored kinematic alignment strategies [35]. This might be a reason why no differences in postoperative PROMs and responder rate could be found in the current study.

This study has several limitations, first of all its retrospective design. Due to the lack of randomization, the results of the analysis are susceptible to a potential selection bias. Cohort matching was not performed. In the study group 77% of patients received navigated TKA, as this is the preferred method in our department. Whenever navigation was not available, for example due to limited capacity of operation theaters with navigational equipment, conventional TKA was performed. Second, measuring mechanical axis in long-leg radiographs is susceptible to measurement errors due to lower limb malrotation [22]. A recently published study has shown a range between 29° of internal rotation and 22° of external rotation, resulting in measurement errors between 0.4° and 1.7° [41]. To minimize measurement errors, the independent examiners followed a strict measurement protocol. Proper lower limb rotation was verified by fibula overlap [23]. Another limitation is the fact that data acquisition was limited to the data available from our institutional joint registry. As a consequence, other parameters with possible influence on outcome, such as pre-operative leg alignment, pre-operative range of motion, BMI, and psychosocial aspects, could not be captured. A further limitation is the limited follow-up time of our study. As data are only available up to one year postoperatively, assessment of long-term outcome and implant durability was not possible. The strength of our study is the higher number of patients in a monocentric study design that guarantees standardized treatment protocols for navigated and conventional TKA. As a consequence, all patients received the same surgical approach, were supplied with the same type of implant and obtained the same standardized postoperative treatment. In this way, possible confounding factors were minimized. Future analyses should focus on long-term results of navigated versus conventional TKA. Although evidence is very limited, navigation could have a positive effect on the long-term revision rate and implant survival [42].

## Conclusion

Both navigated and conventional TKA enables accurate postoperative leg alignmentin a high-volume setting and proved to be safe with regard to periprosthetic fractures, nerve injuries, thromboembolic events and infections. In terms of PROMs and responder rates defined by the OMERACT-OARSI criteria, both navigated and conventional TKA showed good results up to one year postoperatively.

## References

[1] Choi Y-J, Ra HJ (2016). Patient satisfaction after total knee arthroplasty. Knee Surg Relat Res.

[2] (2019) Hip and knee replacement. In: Health at a glance 2019. OECD

[3] Cross M, Smith E, Hoy D (2014). The global burden of hip and knee osteoarthritis: estimates from the Global Burden of Disease 2010 study. Ann Rheum Dis.

[4] Berend ME, Ritter MA, Meding JB (2004). The Chetranjan Ranawat Award: tibial component failure mechanisms in total knee arthroplasty. Clin Orthop.

[5] Fang DM, Ritter MA, Davis KE (2009). Coronal alignment in total knee arthroplasty. J Arthroplasty.

[6] Choong PF, Dowsey MM, Stoney JD (2009). Does accurate anatomical alignment result in better function and quality of life? Comparing conventional and computer-assisted total knee arthroplasty. J Arthroplasty.

[7] Longstaff LM, Sloan K, Stamp N (2009). Good alignment after total knee arthroplasty leads to faster rehabilitation and better function. J Arthroplasty.

[8] Meneghini RM, Grant TW, Ishmael MK, Ziemba-Davis M (2017). Leaving residual varus alignment after total knee arthroplasty does not improve patient outcomes. J Arthroplasty.

[9] Krackow KA, Bayers-Thering M, Phillips MJ (1999). A new technique for determining proper mechanical axis alignment during total knee arthroplasty: progress toward computer-assisted TKA. Orthopedics.

[10] Tsukeoka T, Tsuneizumi Y, Yoshino K (2019). An accelerometer-based navigation did not improve the femoral component positioning compared to a modified conventional technique of pre-operatively planned placement of intramedullary rod in total knee arthroplasty. Arch Orthop Trauma Surg.

[11] Klasan A, Putnis SE, Grasso S (2020). Conventional instruments are more accurate for measuring the depth of the tibial cut than computer-assisted surgery in total knee arthroplasty: a prospective study. Arch Orthop Trauma Surg.

[12] Denti M, Soldati F, Bartolucci F (2018). Conventional versus smart wireless navigation in total knee replacement: similar outcomes in a randomized prospective study. Joints.

[13] Fu Y, Wang M, Liu Y, Fu Q (2012). Alignment outcomes in navigated total knee arthroplasty: a meta-analysis. Knee Surg Sports Traumatol Arthrosc.

[14] Zhu M, Lindsay E, Keenan A (2020). The use of accelerometer-based navigation for coronal TKA alignment: a prospective, single surgeon comparative study. Arch Orthop Trauma Surg.

[15] Rebal BA, Babatunde OM, Lee JH (2014). Imageless computer navigation in total knee arthroplasty provides superior short term functional outcomes: a meta-analysis. J Arthroplasty.

[16] Thienpont E, Fennema P, Price A (2013). Can technology improve alignment during knee arthroplasty. Knee.

[17] Maculé-Beneyto F, Hernández-Vaquero D, Segur-Vilalta JM (2006). Navigation in total knee arthroplasty. A multicenter study. Int Orthop.

[18] Rosenberger RE, Hoser C, Quirbach S (2008). Improved accuracy of component alignment with the implementation of image-free navigation in total knee arthroplasty. Knee Surg Sports Traumatol Arthrosc.

[19] Beldame J, Boisrenoult P, Beaufils P (2010). Pin track induced fractures around computer-assisted TKA. Orthop Traumatol Surg Res.

[20] Marchant DC, Rimmington DP, Nusem I, Crawford RW (2004). Safe femoral pin placement in knee navigation surgery: a cadaver study. Comput Aided Surg.

[21] Moskal J, Capps S, Mann J, Scanelli J (2013). Navigated versus conventional total knee arthroplasty. J Knee Surg.

[22] Moreland JR, Bassett LW, Hanker GJ (1987). Radiographic analysis of the axial alignment of the lower extremity. J Bone Jt Surg Am.

[23] Maderbacher G, Schaumburger J, Baier C (2014). Predicting knee rotation by the projection overlap of the proximal fibula and tibia in long-leg radiographs. Knee Surg Sports Traumatol Arthrosc.

[24] Gastmeier P, Geffers C, Brandt C (2006). Effectiveness of a nationwide nosocomial infection surveillance system for reducing nosocomial infections. J Hosp Infect.

[25] Bellamy N (1989). Pain assessment in osteoarthritis: experience with the WOMAC osteoarthritis index. Semin Arthritis Rheum.

[26] Herdman M, Gudex C, Lloyd A (2011). Development and preliminary testing of the new five-level version of EQ-5D (EQ-5D-5L). Qual Life Res.

[27] Weber M, Craiovan B, Woerner ML (2018). Predictors of outcome after primary total joint replacement. J Arthroplasty.

[28] Pham T, van der Heijde D, Altman RD (2004). OMERACT-OARSI Initiative: osteoarthritis research society international set of responder criteria for osteoarthritis clinical trials revisited. Osteoarthr Cartil.

[29] Tingart M, Lüring C, Bäthis H (2008). Computer-assisted total knee arthroplasty versus the conventional technique: how precise is navigation in clinical routine?. Knee Surg Sports Traumatol Arthrosc.

[30] Dunn OJ (1961). Multiple comparisons among means. J Am Stat Assoc.

[31] Musselwhite C, Holland C, Walker I (2015). The role of transport and mobility in the health of older people. J Transp Health.

[32] Ritter MA, Faris PM, Keating EM, Meding JB (1994) Postoperative alignment of total knee replacement. Its effect on survival. Clin Orthop 153–156 8119010

[33] Werner FW, Ayers DC, Maletsky LP, Rullkoetter PJ (2005). The effect of valgus/varus malalignment on load distribution in total knee replacements. J Biomech.

[34] Mason JB, Fehring TK, Estok R (2007). Meta-analysis of alignment outcomes in computer-assisted total knee arthroplasty surgery. J Arthroplasty.

[35] Courtney PM, Lee G-C (2017). Early outcomes of kinematic alignment in primary total knee arthroplasty: a meta-analysis of the literature. J Arthroplasty.

[36] Xie C, Liu K, Xiao L, Tang R (2012). Clinical outcomes after computer-assisted versus conventional total knee arthroplasty. Orthopedics.

[37] Bauwens K, Matthes G, Wich M (2007). Navigated total knee replacement: a meta-analysis. J Bone Jt Surg.

[38] Graham DJC, Harvie P, Sloan K, Beaver RJ (2011). Morbidity of navigated vs conventional total knee arthroplasty. J Arthroplasty.

[39] Burnett RSJ, Barrack RL (2013). Computer-assisted total knee arthroplasty is currently of no proven clinical benefit: a systematic review. Clin Orthop Relat Res.

[40] Dunbar MJ, Richardson G, Robertsson O (2013). I can’t get no satisfaction after my total knee replacement: rhymes and reasons. Bone Jt J..

[41] Maderbacher G, Matussek J, Greimel F (2019). Lower limb malrotation is regularly present in long-leg radiographs resulting in significant measurement errors. J Knee Sur.

[42] Baier C, Wolfsteiner J, Otto F (2017). Clinical, radiological and survivorship results after ten years comparing navigated and conventional total knee arthroplasty: a matched-pair analysis. Int Orthop.

